# Intestinal Microbiota and Immune Modulation in Zebrafish by Fucoidan From Okinawa Mozuku (*Cladosiphon okamuranus*)

**DOI:** 10.3389/fnut.2020.00067

**Published:** 2020-06-24

**Authors:** Wakako Ikeda-Ohtsubo, Adrià López Nadal, Edoardo Zaccaria, Masahiko Iha, Haruki Kitazawa, Michiel Kleerebezem, Sylvia Brugman

**Affiliations:** ^1^Cell Biology and Immunology Group, Department of Animal Sciences, Wageningen University and Research, Wageningen, Netherlands; ^2^Host-Microbe Interactomics Group, Department of Animal Sciences, Wageningen University and Research, Wageningen, Netherlands; ^3^Laboratory of Animal Products Chemistry, Graduate School of Agricultural Science, Tohoku University, Sendai, Japan; ^4^Livestock Immunology Unit, International Education and Research Center for Food Agricultural Immunology, Graduate School of Agricultural Science, Tohoku University, Sendai, Japan; ^5^South Product Co., Ltd., Uruma, Japan

**Keywords:** fucoidan, immunomodulation, microbiota, seaweed polysaccharides, zebrafish

## Abstract

Fucoidan represents fucose-rich sulfated polysaccharides derived from brown seaweeds, which exerts various biological activities applicable for functional foods and therapeutic agents. The objective of the present study was to investigate *in vivo* effects of fucoidan extracted from Okinawa mozuku (*Cladosiphon okamuranus*), common edible seaweed in Japan, on immune responses and microbiota composition in zebrafish. We treated larvae and adult zebrafish with Okinawa mozuku (OM) fucoidan by immersion (100 and 500 μg/mL, 3 days) and by feeding (3 weeks), respectively. The effect of OM fucoidan on immune responses in zebrafish larvae was evaluated by live imaging of neutrophils and macrophages as well as quantitative polymerase chain reaction of pro- and anti-inflammatory cytokine genes. Whole microbiota of zebrafish larvae and intestinal microbiota of adult zebrafish treated with OM fucoidan were analyzed by Illumina MiSeq pair-end sequencing of the V3–V4 region of 16S rRNA genes. Fucoidan treatment only slightly affected the composition of the larvae microbiota and the number of neutrophils and macrophages, while pro- and anti-inflammatory cytokine gene expression levels were upregulated in the larvae treated with 500 μg/mL OM fucoidan. In contrast, feeding of OM fucoidan clearly altered the intestinal microbiota composition of adult zebrafish, which was characterized by the emergence and predominance of multiple bacterial operational taxonomic units (OTUs) affiliated with Rhizobiaceae and Comamonadaceae at the expense of *E. coli*-related Enterobacteriaceae, the dominant OTUs throughout the studied samples. These changes were accompanied by decreased expression levels of pro-inflammatory cytokine *il1b* in the intestines of the adult zebrafish. Our current study provides the first insights into *in vivo* modulatory effects of fucoidan on microbiota and immune responses of unchallenged zebrafish, which underscores the potential of fucoidan to play a modulatory role in the diet–microbiota–host interplay.

## Introduction

Fucoidan represents polysaccharides consisting of α-(1 → 3) or α-(1 → 4) -linked l-fucose residues with sulfate substitutions, which occasionally contain acetate, glucuronic acid, and monosaccharides such as mannose and galactose (1). Fucoidan from different algal origins has been reported to exhibit unique properties such as antiinflammatory, antiallergic, antitumor, or antiviral effects (2, 3) and is therefore recognized as a prospective ingredient for functional foods and for therapeutic agents (3, 4). Although beneficial effects of fucoidan have been well-studied and described, daily intake of fucoidan from brown seaweed is still not common in Western countries. In Japan, daily seaweed consumption can exceed ~5 g/day (5) and the brown seaweed *mozuku* represents one of the most common edible seaweeds, which is usually consumed raw. Okinawa mozuku (*Cladosiphon okamuranus*) is exclusively cultivated and used in the traditional cuisine on the Okinawan Islands in Japan, a region that is well-known for its high prevalence of centenarians and the general healthy states of its elderly population (6). Fucoidan extracted from Okinawa *mozuku* (OM fucoidan) has a simple structure with a backbone of α-(1 → 3) fucopyranose, substituted with sulfate and α-glucuronic acid at ~50 and 17% of its residues, respectively (7). Similar to what has been shown for fucoidan derived from other origins, OM fucoidan has been reported to exert antitumor and antiviral effects. In a murine model, antitumor activity has been attributed to the fucoidan-mediated stimulation of macrophages and natural killer cells (8, 9), while antiviral activities seem to be more complex and may involve both host–virus and virus–fucoidan interactions. Previous studies have reported antiviral activities of OM fucoidan against human T-cell leukemia virus type 1 (HTLV-1) (10, 11), dengue virus type 2 (12), hepatitis C (13), Newcastle disease virus (DSV) in poultry (14, 15), and canine distemper virus (CDV) (16). Collectively, these studies support the high potential of OM fucoidan as a therapeutic agent in viral infections.

Meanwhile, effects of OM fucoidan on the intestinal microbiota remain poorly understood. Polysaccharides such as fucoidan have a potential to not only mechanistically interfere with host–microbiota interactions but also to serve as nutrition for bacteria constituting the microbiota (17, 18). Since no enzymes digesting fucoidan have been found in animal intestinal tracts, fucoidan can reach the lower intestinal tract intact and may confer beneficial effects on microbiota as prebiotics (19–21). Importantly, some studies have suggested that bioactivities of fucoidan may be attributable to its modulatory effects on gut microbiota. A recent study has shown that fucoidan from *Undaria pinnatifida* can affect host lipid metabolism by modulating the gut microbiota composition (22), which may also explain the effect of OM fucoidan to ameliorate dyslipidemia in rodents (23). Other studies have reported that fucoidan from sea cucumber (*Acaudina molpadioides*) and *hijiki* seaweed (*Sargassum fusiforme*) can relieve symptoms of diabetes by modulating gut microbiota (24, 25).

Considering possible interactions between microbiota and host immune responses, it is crucial to evaluate host immunity and microbiota simultaneously to elucidate the prebiotic potential OM fucoidan (18). Zebrafish offer an ideal *in vivo* model to investigate how fucoidan affects host immunity and microbiota under normal (unchallenged) conditions because of their compatibility with live visualization (26). Using a double-transgenic zebrafish model combined with next-generation sequencing of 16S rRNA genes, we have recently shown that microbiota modulation by antibiotics can significantly affect host inflammatory immune responses in zebrafish larvae immersed in saponin (27). In this study, we exploited this approach to investigate how OM fucoidan can affect immune response and microbiota composition of zebrafish larvae. We also investigated the effect of OM fucoidan on immune responses and intestinal microbiota of adult zebrafish, which were fed with OM fucoidan for 3 weeks.

## Materials and Methods

### Ethics Statement

The present study was approved by the Dutch Committee on Animal Welfare and the Animal Welfare Body (IvD) of Wageningen University, The Netherlands. Furthermore, we adhere to our standard biosecurity and institutional safety procedures at Wageningen University and Research.

### Zebrafish and Fucoidan

Tg (mpeg1:mCherry/mpx:eGFPi^114^) (28, 29) and wild-type zebrafish were maintained in Zebtec family tanks (Tecniplast, Buguggiate, Italy) under continuous flow-through at 28°C (14/10-h light/dark cycle) and fed daily with Tetramin Flakes (Tetra, Melle, Germany). For the experiments using zebrafish larvae, embryos were obtained from the adult transgenic zebrafish by natural spawning and raised with embryo medium (E3) water as described previously (27). OM fucoidan powder (>95% pure fucoidan) extracted from *C. okamuranus* as described previously (30) was provided by South Product Co., Ltd., Okinawa, Japan. The characteristics of this fucoidan were as follows: average molecular weight of 49.8 kDa, l-fucose content of 52.7%, uronic acid content of 18.0%, and sulfate ion content of 17.6%. The OM fucoidan powder was stored at room temperature until use. Fucoidan treatment of larvae and adult zebrafish was performed as follows: the zebrafish larvae (3 days post fertilization; dpf) were randomly distributed in six-well plates (*n* = 8 fish/well) and kept in different concentrations (0, 100, and 500 μg/mL of E3 water) of OM fucoidan (immersion) until 6 dpf. Ten adult zebrafish were maintained in two separate tanks in a continuous flow and temperature-controlled (28°C) system and fed once daily with Tetramin Flakes (control group) or a combination of the flakes and OM fucoidan at the ratio of 1:1 (fucoidan group) over 3 weeks.

### *In vivo* Imaging of Neutrophils and Macrophages in Zebrafish Larvae

Tg (mpeg1:mCherry/mpx:eGFPi^114^) zebrafish larvae were anesthetized with MS-222 (tricaine methane sulfonate) solution and embedded in 1% low melting point agarose (Thermo Fisher Scientific, Waltham, MA, USA), as previously described (27). Larvae were imaged as whole mounts with a Leica M205 FA Fluorescence Stereo Microscope. Neutrophils and macrophages in the intestinal region of each specimen were quantified by counting the total number of cells per defined area using the cell counter plugin available in ImageJ® software (31).

### Relative Gene Expression by Quantitative Polymerase Chain Reaction

Zebrafish were euthanized with MS-222 and the whole larvae (five or six fish per 1.5-mL tube) were preserved in RNA later™ at −20°C. Adult zebrafish were anesthetized with MS-222 and intestines were isolated by dissection and were preserved in RNA later™ at −20°C. Total RNA was isolated from larvae or intestinal samples from adult zebrafish using the RNeasy® Micro Kit (QIAGEN, Venlo, The Netherlands) according to the manufacturer's instructions. After quantifying RNA by a NanoDrop 1000 Spectrophotometer (Thermo Fisher Scientific, Waltham, MA, USA), cDNA was generated from 1 μg of RNA using Superscript™ III First Strand Synthesis Systems (Invitrogen, Carlsbad, CA, USA) according to the manufacturer's instructions. The diluted cDNA corresponding to 125 ng of RNA was used as a template for each reaction of quantitative polymerase chain reaction (qPCR) using the ABsolute^TM^ qPCR SYBR® Green Mix (Thermo Fisher Scientific, Waltham, MA, USA) as previously described (27). The sequences of the primer used in this study can be found in Table S1. The amplification data of each sample were normalized to the reference gene *elf1*α and calculated using the Pfaffl quantification method with efficiency correction (32), as described by Forlenza et al. (33). The statistical significance of differences between the control and fucoidan-treated groups was assessed by a one-way analysis of variance (ANOVA) test using R version 3.5.3 (34) where <0.05 was regarded as significant.

### 16S rRNA-Based Analyses of Zebrafish Microbiota

Zebrafish were euthanized with MS-222 and the whole larvae (four fish per 1.5-mL tube) were washed with sterilized phosphate-buffered saline and preserved at −20°C. Adult zebrafish were euthanized with MS-222 and the intestines were isolated by dissection. The intestinal contents were preserved in InhibitEX Buffer supplied in the QIAamp® DNA Fast Stool Mini Kit (QIAGEN, Venlo, The Netherlands) at −20°C. Total DNA was isolated from the whole larvae or intestines of adult zebrafish using the QIAamp® DNA Fast Stool Mini Kit according to the manufacturer's instructions. Pair-end sequencing was performed using Illumina MiSeq (BaseClear, Leiden, The Netherlands) using amplicons generated with the primer pair 341F−785R that target the V3–V4 variable region of the 16S rRNA gene of most bacteria (35). Raw Illumina sequencing reads were pair-ended, end-trimmed, filtered, and clustered into operational taxonomic units (OTUs) using the microbial genomic module 3.0 implemented in the CLC Bio Genomics Workbench v7.5.1 (Qiagen, Venlo, The Netherlands), 16S Microbiome Pipeline in the EZBioCloud web server (36), or the MICCA pipeline (37), for which OTU assignment was performed using the SILVA ribosomal RNA reference database [release 128, 97% similarity threshold, (38)], the EZBioCloud database (36), and the Ribosomal Database Project (RDP) classifier [version 2.11, 97% identity threshold, (39)], respectively. After confirming the reproducibility of the core microbiota composition of each sample, OTU tables in BIOM format generated by the CLC Bio Genomics Workbench was used for statistical analyses of the diversity and richness (alpha- and beta-diversity) implemented in MicrobiomeAnalyst© using the default filtering parameter settings (40). Significantly different taxa between control and fucoidan-treated group were identified by differential abundance (DESeq2) analysis by R version 3.5.3 (34) and by Linear Discriminant Analysis Effect Size (LEfSe) analysis implemented with EZBioCloud (36, 41).

### Statistical Analysis

The quantified data collected from the fluorescent *in vivo* imaging of the zebrafish larvae and qPCR was analyzed using Student's *t*-test assuming unequal variation as well as one-way analysis of variance (ANOVA) test using Microsoft Excel® and R version 3.5.3 (34), where <0.05 was regarded as significant. The indices of α-diversity and β-diversity for comparing compositional structure of microbiota of each larva and adult zebrafish group were calculated on a species-level summarization of the rarefied OTU tables generated as described in the preceding text. Chao1 and Abundance-based Coverage Estimator (ACE) as well as Shannon and Simpson indices were used to measure the species-level community richness and species level community evenness, respectively, and each index was calculated using the online module of Microbiomeanalyst© (40). The plots of β-diversity indicating dissimilarities between samples were produced by principal coordinates analyses (PCoA) calculated using the Bray–Curtis dissimilarity index implemented in Microbiomeanalyst© (40).

## Results

### Effect of OM Fucoidan on Innate Immunity of Zebrafish Larvae

**Figure 1:**
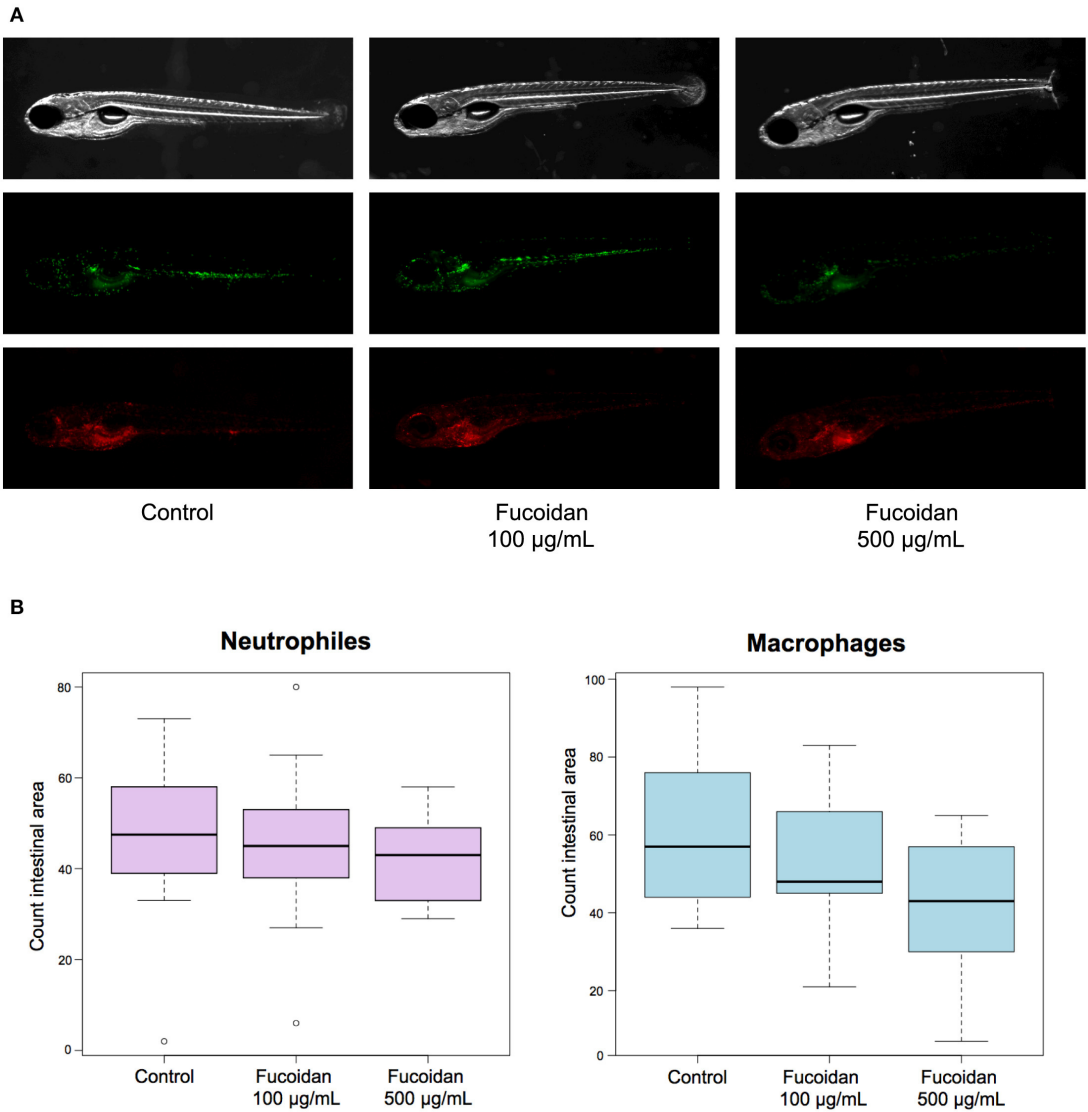
Effect of OM fucoidan on lymphocyte recruitment to the intestinal region of zebrafish larvae. **(A)** Representative pictures of 6 dpf zebrafish larvae (mpeg:mCherry, mpx:GFP) displaying neutrophils (green) and macrophages (red). F100, the zebrafish larvae treated with 100 μg/mL OM fucoidan from 3 dpf for 3 days; F500, the zebrafish larvae treated with 500 μg/mL OM fucoidan from 3 dpf for 3 days. **(B)** Quantification of neutrophils and macrophages in the intestinal area of larval zebrafish. Control, no treatment (*n* = 14); F100, treated with 100 μg/mL (*n* = 13); F500, treated with 500 μg/mL (*n* = 17).

Zebrafish larvae (3−6 dpf) treated with OM fucoidan (0, 100, and 500 μg/mL of E3 water) by immersion showed normal development without visible signs of damage relative to untreated controls (data not shown). To investigate whether the treatment with OM fucoidan affected cellular immunity of zebrafish larvae, the numbers of neutrophils (mpx:GFP) and macrophages (mpeg1:mCherry) in the intestinal area of the control and the fucoidan-treated larvae were compared. There was no observable difference between the live-imaged control and the fucoidan-treated larvae (Figure 1A). The cell counts of neutrophils and macrophages in fucoidan-treated zebrafish larvae tended to be reduced compared to the control, but the difference was not significant (*P* > 0.1, Figure 1B).

**Figure 2:**
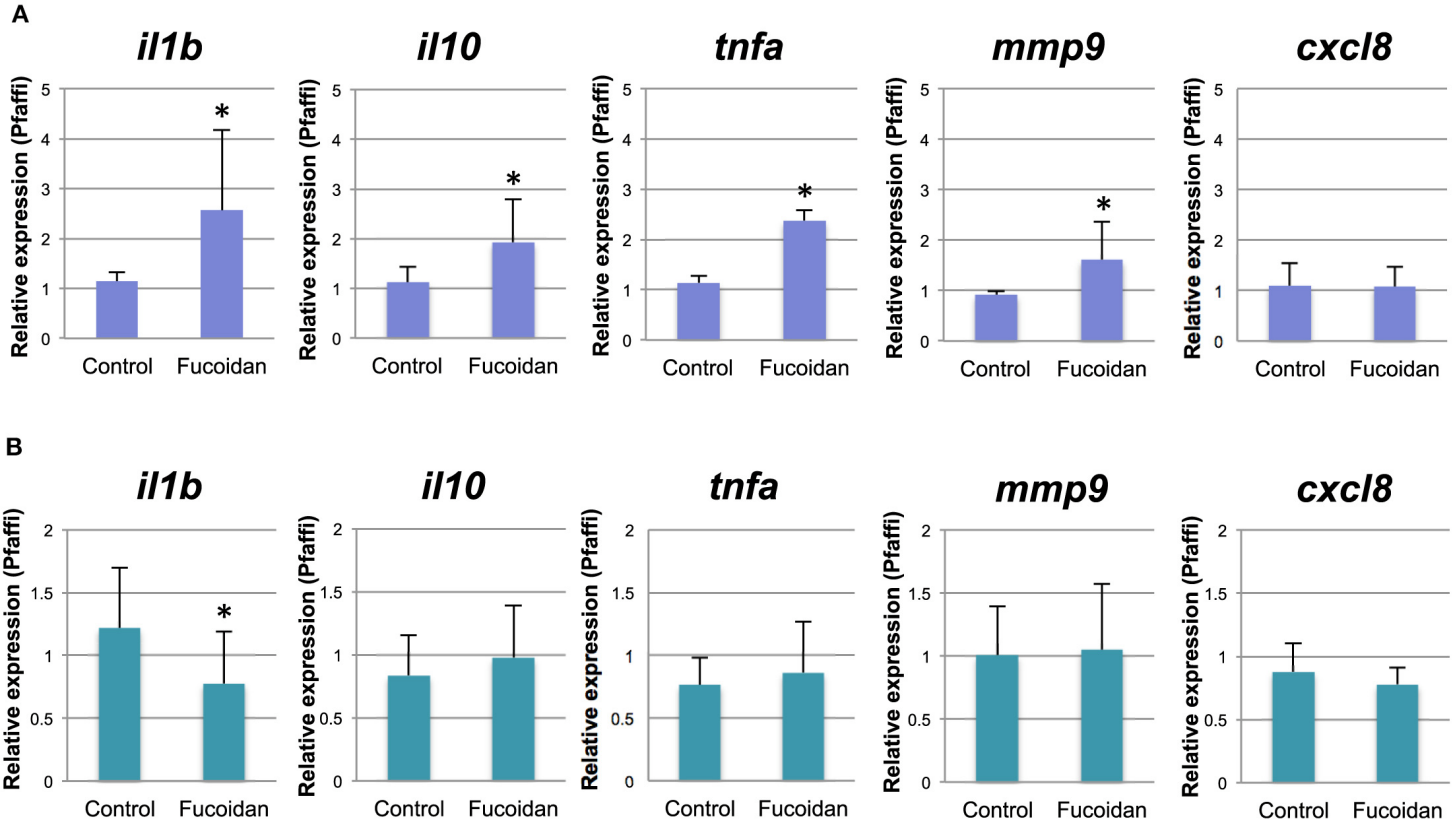
Relative gene expression of immune genes of zebrafish treated with OM fucoidan. **(A)** Control, 6 dpf zebrafish larvae; Fucoidan, 6 dpf zebrafish larvae immersed in 500 μg/mL fucoidan from 3 dpf for 3 days. **(B)** Control, intestinal samples of adult zebrafish fed with standard fish meal for 3 weeks; Fucoidan, intestinal samples of adult zebrafish fed with OM fucoidan meal (Tetramin fish flakes: OM fucoidan = 1:1). The asterisk denotes significant differences from the control samples (*P* < 0.05, 1-way ANOVA).

Using the same experimental setup, we performed qPCR to measure the relative expression levels of selected genes representing host immune cell responses in the fucoidan (500 μg/mL) -treated zebrafish larvae and the untreated control. The relative gene expression levels of pro- (*il1b, tnfa*) and antiinflammatory (*il10*) cytokines as well as *mmp9* were moderately (1.7–2.2 fold) upregulated in the larvae treated with 500 μg/mL of fucoidan (Figure 2A). No significant difference was observed for *cxcl-8a* (Figure 2A) and gene transcripts for *il-17f* , *il-22*, and *tnfb* were not detected in our samples (data not shown).

Adult zebrafish fed with OM fucoidan for 3 weeks did not show visible changes in fitness and behavior compared to controls fed the Tetramin Flakes only (data not shown). Immune responses of the adult zebrafish intestines were examined by quantitative PCR using primers specific for *il1b, il-10, cxcl-8a, tnfa*, and *mmp9*. Overall, there was no significant difference between the fucoidan-fed zebrafish and the control, except for *il1b*, which was expressed at slightly decreased levels (0.63-fold) in the fucoidan-fed zebrafish compared to the control (Figure 2B).

### Effect of OM Fucoidan on Microbiota Diversity and Composition of Larval and Adult Zebrafish

The effects of OM fucoidan on diversity of larval and adult zebrafish microbiota were analyzed by 16S rRNA gene amplicon sequencing. Whole-body DNA samples of pools of 6 dpf zebrafish larvae were obtained from the three groups (*n* = 4 per group; immersion in 0, 100, and 500 μg/mL of OM fucoidan in E3 water) and used for Illumina MiSeq sequencing of 16S rRNA genes. For adult zebrafish, intestinal DNA from each of the two groups (*n* = 5 per group; control vs. OM-fucoidan fed) was used. A summary of the sequencing results is shown in Table S2 and the rarefaction curves of all samples, except one sample (WO3, a control sample of zebrafish larvae), reached saturation (Figure S1).

**Figure 3:**
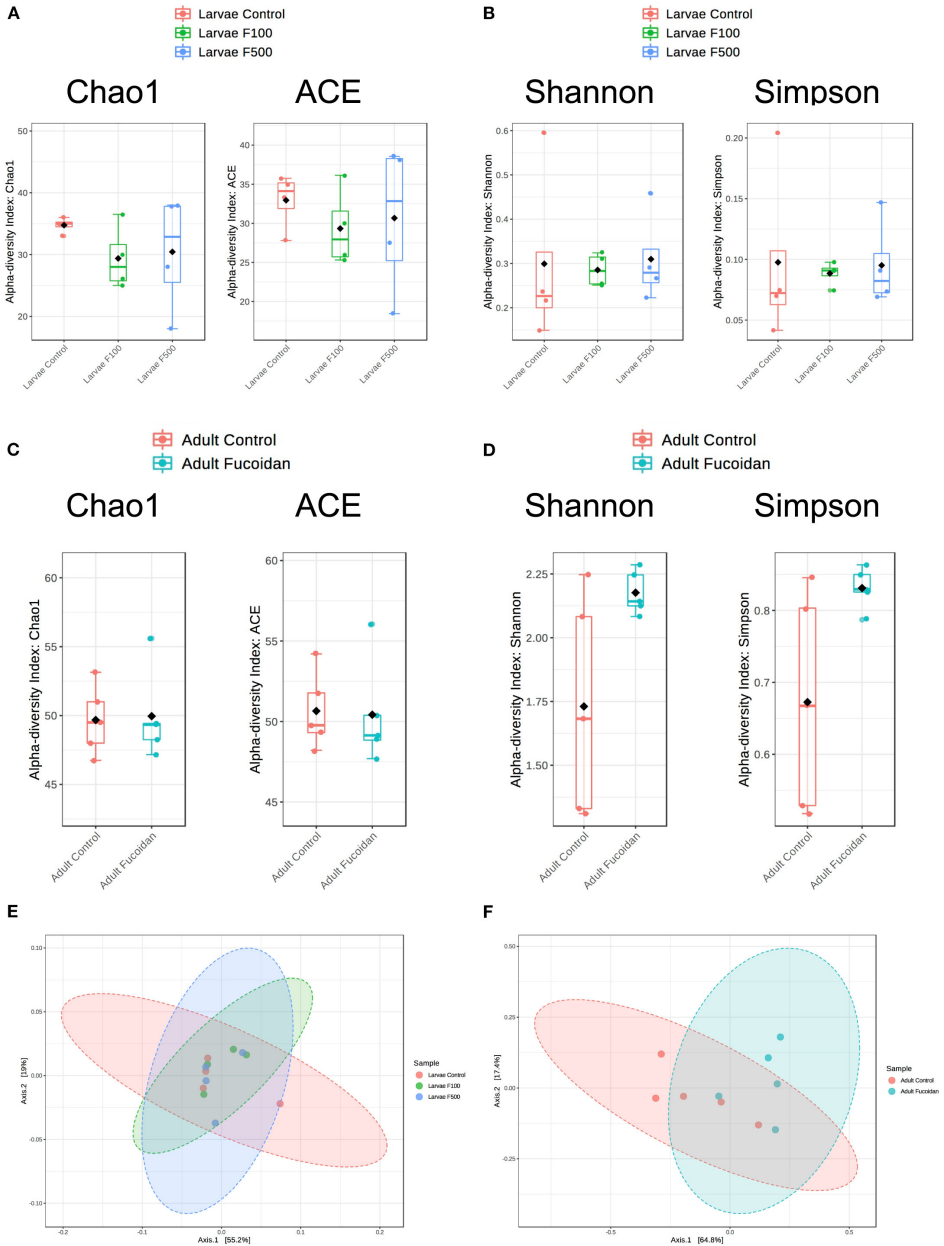
Effect of OM fucoidan on the diversity of larvae zebrafish microbiota and adult zebrafish intestinal microbiota. Species-level community richness **(A,C)** and species level community evenness **(B,D)** were compared between larvae zebrafish samples (**A**, **B**, **E**; F100, the zebrafish larvae treated with 100 μg/mL OM fucoidan from 3 dpf for 3 days; F500, the zebrafish larvae treated with 500 μg/mL OM fucoidan from 3 dpf for 3 days) and adult zebrafish intestinal samples (**C**, **D**, **F**; Fucoidan [Tetramin fish flakes: OM fucoidan = 1:1] for 3 weeks). Beta-diversity of larval **(E)** and adult intestinal **(F)** microbiota compared by the principal coordinates analyses (PCoA) based on Bray–Curtis dissimilarity index.

In microbiota of zebrafish larvae, no significant differences were observed in the species richness (Chao1, *P* = 0.47265; ACE; *P* = 0.74339; Figure 3A) or the species evenness (Shannon, *P* = 0.96621; Simpson, *P* = 0.96058; Figure 3B) between the control and fucoidan-treated fish, regardless of the fucoidan concentrations. In intestinal microbiota of adult zebrafish, the species richness was also not affected (Chao1, *P* = 0.88482; ACE; *P* = 0.90299; Figure 3C), while the species evenness tended to be moderately increased in the fucoidan-fed zebrafish (Shannon, *P* = 0.079088; Simpson, *P* = 0.078456; Figure 3D). β-Diversity analyses showed no significant association between fucoidan treatment and microbiota composition of zebrafish larvae (*P* < 0.49845; Figure 3E), which was in contrast to adult zebrafish, in which fucoidan-feeding was moderately associated with changes in the species composition of intestinal microbiota (*P* < 0.023, Figure 3F). Collectively, these results indicate that the treatments with OM fucoidan affected the diversity and composition of intestinal microbiota of adult zebrafish, but not the larvae zebrafish microbiota.

**Figure 4:**
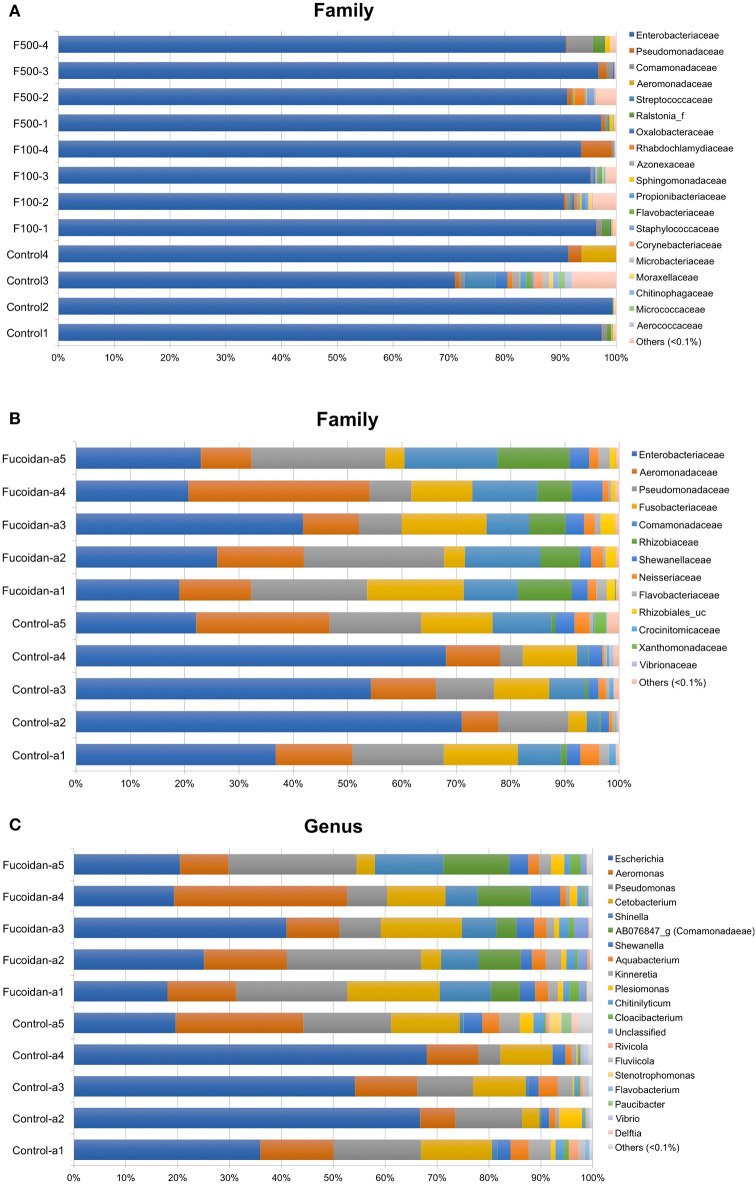
Effects of OM fucoidan on the bacterial community structure of larvae zebrafish microbiota and adult zebrafish intestinal microbiota. **(A)** Composition of family-level bacterial groups in the larvae zebrafish microbiota (F100, the zebrafish larvae treated with 100 μg/mL OM fucoidan from 3 dpf for 3 days; F500, the zebrafish larvae treated with 500 μg/mL OM fucoidan from 3 dpf for 3 days.). **(B)** Composition of family-level bacterial groups in the adult zebrafish intestinal microbiota. **(C)** Composition of genus-level bacterial groups in adult zebrafish intestinal microbiota. **(B,C)** Fucoidan [Tetramin fish flakes: OM fucoidan = 1:1] for 3 weeks). The taxonomic assignment is based on the latest EZbioCloud database (36). Taxa representing <0.1% of the total community are not visualized.

Taxonomic assignment of OTUs generated from each sample was performed using EzBioCloud database (https://www.ezbiocloud.net), which offers a high genus and species-level resolution (36). Consistent with our previous work (27), microbiota of zebrafish larvae was predominated (>95%) by Enterobacteriaceae (Figure 4A), which were affiliated with the *Escherichia coli* group (Figure S2, Table S3). Consistent with the diversity analyses (Figure 3), the relative abundance of each bacterial species and OTUs were similar between the larvae samples and did not reflect an effect of OM fucoidan (Figure 4A, Figure S2). In addition, DESeq2 and LEfSe analyses failed to identify significantly different taxa between the control and fucoidan-treated zebrafish larvae.

**Figure 5:**
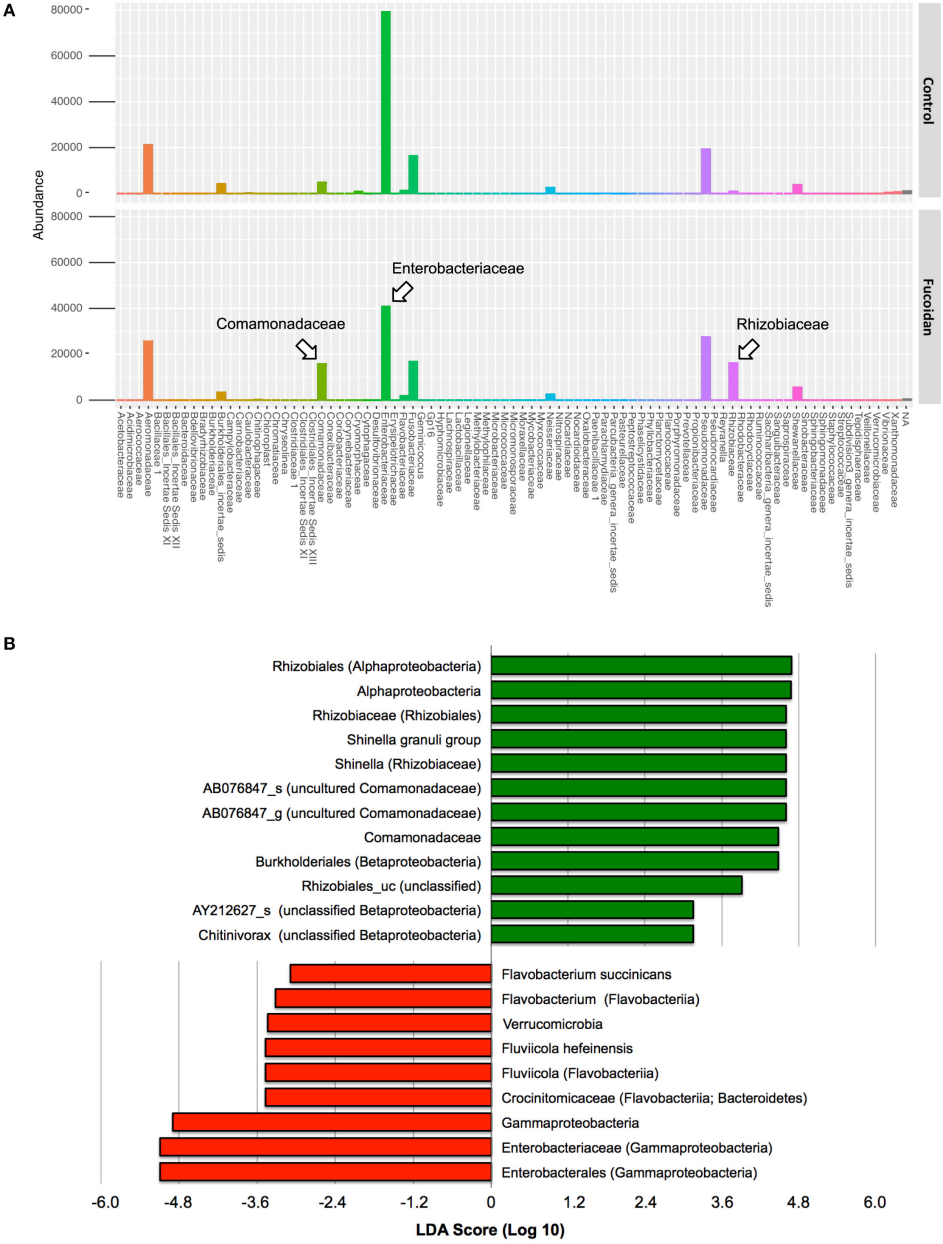
Significantly different taxa in the adult zebrafish intestinal microbiota associated with the OM fucoidan treatment. **(A)** Differently abundant bacterial families between the intestinal microbiota of the control and OM fucoidan-fed adult zebrafish, identified by differential abundance analysis using DESeq2 in R. The taxonomy assignment of the OTU dataset used is based on the RDP classifier (version 2.11, 97% identity threshold, 39). **(B)** Specific bacterial groups positively (green)- and negatively (red)- associated with OM fucoidan treatment. Identification of the significantly different taxa and LDA score calculation were performed by Linear discriminant analysis effect size (LEfSe) tool implemented with EZBioCloud (36). The taxonomic assignment is based on the latest EZbioCloud database (36).

In contrast, a significant difference was observed in the intestinal microbiota between the control and fucoidan-fed adult zebrafish (Figures 4B,C, Figure S3). The class to species-level composition of intestinal microbiota of fucoidan-fed zebrafish was clearly different from the control (Figure S3), and this difference is characterized by the emergence and increase of relative abundance of several bacterial groups affiliated with Comamonadaceae and Rhizobiaceae (Figure 4B, Figure S3). At the genus and species level, these families were represented by unclassified genus of Comamonadaceae [AB076847, (42)] and the genus *Shinella granuli* group (Rhizobiaceae) (Figure 4C, Figure S3). The predominant families Comamonadaceae and Rhizobiaceae were also identified as the significantly different taxa represented in the fucoidan-fed zebrafish by the DESeq2 and LEfSe analyses (Figures 5A,B). Interestingly, both analyses revealed that the increase of Comamonadaceae and Rhizobiaceae were concomitant with the decrease of Enterobacteriaceae (Figures 5A,B, Figure S3). LEfSe analysis also found that unclassified groups of Rhizobiales and Betaproteobacteria were significantly associated with the fucoidan-feeding, while Flavobacteriia (phylum Bacteroides) were negatively affected (Figure 5B).

## Discussion

Our current study showed that fucoidan derived from Japanese brown seaweed *C. okamuranus* has the potential to modulate the intestinal microbiota of adult zebrafish. The profound compositional change associated with the fucoidan-feeding in adult zebrafish can be characterized by the increased abundance of bacterial groups affiliated with Comamonadaceae and Rhizobiaceae and a decreased abundance of Enterobacteriaceae. Although non-pathogenic bacteria of the *E. coli* species have been proposed to confer a protective effect on zebrafish larvae via lipopolysaccharide (LPS) tolerance and acid production (43, 44), numerous studies have reported proinflammatory effects of Enterobacteriaceae in fish (45, 46). In addition, Enterobacteriaceae are thought to be responsible for the spread of antimicrobial resistance in aquatic environments (47). In this context, it is intriguing that the relative expression of *il1b*, a proinflammatory cytokine, was moderately downregulated in the fucoidan- fed adult zebrafish (Figure 2B), which suggests that OM fucoidan may have directly or indirectly suppressed the dominance of Enterobacteriaceae that can induce proinflammatory responses. This type of diet–microbiota–host interplay is likely to play a crucial role in pro- and antiinflammatory states in animal intestines [(48) for review], and it is therefore of great interest further investigate in future studies on the mechanism that OM fucoidan decreases the relative abundance of Enterobacteriaceae in the fish intestine with regard to how it may impact the health of the fish population.

While metabolic and physiological properties of the intestinal bacteria of adult zebrafish that responded to the fucoidan feeding are yet to be determined, the increase of Comamonadaceae and Rhizobiaceae suggests their involvement in the degradation of OM fucoidan. Interestingly, Comamonadaceae and Rhizobiaceae have been frequently found in a nitrogen removal process in wastewater treatment systems called solid-phase denitrification (49), where solid biodegradable polymers are used as carbon sources for denitrifying bacteria (50). This system is also applicable for nitrogen removal in aquaculture, where increased nitrate concentration poses negative effects on fish (51). Indeed, the unclassified Comamonadaceae [AB076847, (42)] and *Shinella* spp., the intestinal abundance of which increased in response to the fucoidan feeding of adult zebrafish in our current study, have been reported to belong to denitrifying bacteria possessing biodegrading abilities of diverse compounds including biopolymers and xenobiotics (42, 52, 53). Therefore, it seems plausible that OM fucoidan may serve as a carbon source for intestinal bacteria of adult zebrafish, and identification of the degradation pathways involved awaits further investigation

Although a large body of studies has documented that the host innate immunity plays significant roles for shaping microbiota and vice versa (26, 54), our current results imply that the compositional structure of microbiota is not strongly correlated to the expression patterns of host immune genes. Feeding of OM fucoidan for 3 weeks profoundly altered gut microbiota composition in adult zebrafish; however, the analysis of a selected set of immune genes only showed a slight reduction in the expression of *il1b*. In contrast, the exposure of zebrafish larvae to OM fucoidan resulted in changes in expression of immune genes (*il1b, il10, tnfa*, and *mmp9*) but did not affect the microbiota composition. These results might be partly explained by the timing and duration of the exposure. Since larval feeding starts in the immersion window, it is expected that the immune system of these developing larvae is responding to novel antigens that it is exposed to. In contrast, in adult fish, the immune system has fully developed and a proper homeostasis is reached at the mucosal surfaces such as the intestines (55). Furthermore, since we only evaluated the immune response in the intestines at 3 weeks after feeding, the initial immune modulatory effect of primary exposure to fucoidan might have been missed, while the microbiota had 3 weeks to adapt to the new substrate provided. Future studies will include multiple time points to address early vs. late immune modulatory effects at different time points in life (of fish).

Furthermore, as has been shown in a study by Burns et al. using innate immune-deficient Myd88 knockout zebrafish (56), the gut microbiota composition can be better explained by the interhost dispersal effect, i.e., transmission and sharing microbiota among hosts, than immune gene expression patterns. Also, Stagaman et al. have reported that the effects of adaptive immunity on microbiota composition can be overwhelmed by other factors derived from co-housing within the same tank (57). Our study also reflected this phenomenon, since all adult zebrafish fed with OM fucoidan showed the same compositional changes in the relative abundance of specific bacterial groups (Figures 4B,C, Figure S3). The interhost dispersal of Comamonadaceae and Rhizobiaceae among adult zebrafish associated with the fucoidan-feeding suggests that these specific bacterial groups are subject to filtering by local host environments. Further studies are warranted to determine whether the interhost dispersal and OM fucoidan reciprocally affected the microbial composition.

In contrast to previous studies reporting inhibitory effects of fucoidan on inflammatory responses of injury zebrafish models (58, 59), the influence of OM fucoidan treatments on the baseline zebrafish immune responses were rather mild in our current study. In addition, while a previous study has implied a high concentration of fucoidan may be cytotoxic (60), immersion of zebrafish larvae (3–6 dpf) in OM fucoidan at concentrations of 100 and 500 μg/mL did not affect their fitness. In previous studies using LPS-challenged zebrafish models (58, 59), the antiinflammation effects of fucoidan may be rather explained by the interference of LPS–host interaction rather than direct modulation of host immunity. Another interpretation of the less profound effect on the baseline immune response of zebrafish to fucoidan is that zebrafish immunity may have evolved to become tolerant to the constituents of blown algae abundant in their original habitats (61).

Our current finding that OM fucoidan modulated the gut microbiota composition of zebrafish is in line with studies using rodents (22, 23). The improvement of diabetic symptoms attributed to the modulation of gut microbiota by fucoidan that have been shown in previous studies (24, 25) implies that fucoidan feeding and the subsequent alteration of intestinal microbiota may also affect metabolic properties of fish. Future studies toward a better understanding of the commonalities between intestinal microbial metabolism and host responses shared by fish and animals (62, 63) will help us to evaluate the potential of OM fucoidan as a new prebiotic in aquaculture (64).

## Conclusions

Treatment with OM fucoidan moderately modulated the relative expression of innate immune genes in larvae zebrafish, while no change in microbiota composition was observed. In adult zebrafish, feeding OM fucoidan increased the relative abundance of Comamonadaceae and Rhizobiaceae at the expense of Enterobacteriaceae, which was accompanied by a slight decrease of relative expression of a proinflammatory gene *il1b*, which suggests a potential of OM fucoidan to shift the microbial composition to an antiinflammatory state by selectively suppressing populations of bacteria that are associated with proinflammatory responses. To our knowledge, this is the first study to describe *in vivo* modulatory effects of fucoidan on microbiota and immune responses of unchallenged zebrafish.

## Data Availability Statement

The raw sequence data files were deposited in the NCBI sequence read archive (SRA) database under BioProject number PRJNA597701 (SRA accession numbers listed in Supplementary File “data sheet 3.xlsx”).

## Ethics Statement

The animal study was reviewed and approved by Dutch Committee on Animal Welfare (2017.W-0034) and the Animal Welfare Body (IvD) of the Wageningen University (Netherlands).

## Author Contributions

WI-O, AL, MK, and SB designed the research. WI-O, AL, and SB conducted the experiments. WI-O, AL, EZ, MK, and SB analyzed the data. WI-O and SB wrote the article. MK and SB provided the funding. All authors critically reviewed the manuscript and approved the manuscript.

## Conflict of Interest

MI is employed by the company South Product Co., Ltd. The remaining authors declare that the research was conducted in the absence of any commercial or financial relationships that could be construed as a potential conflict of interest.
