# Supplementary material for: Intestinal Microbiota and Immune Modulation in Zebrafish by Fucoidan From Okinawa Mozuku (Cladosiphon okamuranus)
Source: Front Nutr. 2020 Jun 24;7:67. doi: 10.3389/fnut.2020.00067 (PMC7327095; doi:10.3389/fnut.2020.00067)
Supplement: Supplementary file 1 [file Data_Sheet_1.PDF]

SUPPLEMENTARY MATERIAL

# Modulation of immune responses and intestinal microbial composition in zebrafish by fucoidan derived from Okinawa *mozuku* (*Cladosiphon okamuranus*)

**Wakako Ikeda-Ohtsubo, Adrià López Nadal, Edoardo Zaccaria, Masahiko Iha, Haruki Kitazawa, Michiel Kleerebezem, Sylvia Brugman**

Supplementary Table 1. Sequence of oligonucleotide primers used in real-time qPCR used in this study

| Gene name | Forward Primer (5'-3') | Reverse Primer (5'-3') |
|---|---|---|
| *elfα1* | CTGGAGGCCAGCTCAAACAT | ATCAAGAAGAGTAGTAGTACCG |
| *il1β* | TGCGGGCAATATGAAGTCA | TTCGCCATGAGCATGTCC |
| *il10* | AGGGCTTTCCTTTAAGACTG | TTCGCCATGAGCATGTCC |
| *clcl8a* | TGTTTTCCTGGCATTTCTGACC | TTTACAGTGTGGGCTTGGAGGG |
| *tnfα* | CAGGGCAATCAACAAGA | CCTGGTCCTGGTCATCT |
| *tnfβ* | AAACAACAAATCACCACACC | ACACAAAGTAAAGACCATCC |
| *mmp9* | ACGGCATTGCTGACAT | TAGCGGGTTTGAATGG |
| *il17* | AACCGGTTGTGTGATACTG | CTGGGCTTCAAAGATGAC |
| *il22* | GGAGGGTCTGCACAGAG | TAGCGGGTTTGAATGG |

Supplementary Table 2. Summary of Illumina Miseq sequencing reads

| Sample ID | Fish | Treatment | Total number of reads | No. of valid reads for OTU clustering | No. of species found (EZBioCloud) |
|---|---|---|---|---|---|
| WO1 | Larvae | Control | 34,295 | 30,752 | 64 |
| WO2 | Larvae | Control | 35,664 | 31,940 | 56 |
| WO3 | Larvae | Control | 15,484 | 6,788 | 124 |
| WO4 | Larvae | Control | 31,317 | 28,119 | 58 |
| WO5 | Larvae | Fucoidan (100 µg/ml) | 35,533 | 31,659 | 72 |
| WO6 | Larvae | Fucoidan (100 µg/ml) | 23,856 | 19,935 | 59 |
| WO7 | Larvae | Fucoidan (100 µg/ml) | 25,129 | 21,708 | 54 |
| WO8 | Larvae | Fucoidan (100 µg/ml) | 27,998 | 25,187 | 54 |
| WO9 | Larvae | Fucoidan (500 µg/ml) | 41,070 | 36,810 | 72 |
| WO10 | Larvae | Fucoidan (500 µg/ml) | 10,952 | 8,754 | 68 |
| WO11 | Larvae | Fucoidan (500 µg/ml) | 33,106 | 29,792 | 42 |
| WO12 | Larvae | Fucoidan (500 µg/ml) | 39,512 | 34,300 | 75 |
| WO13 | Adult | Control (intestine) | 36,761 | 31,419 | 74 |
| WO14 | Adult | Control (intestine) | 36,982 | 32,874 | 65 |
| WO15 | Adult | Control (intestine) | 35,574 | 30,818 | 116 |
| WO16 | Adult | Control (intestine) | 29,552 | 25,857 | 79 |
| WO17 | Adult | Control (intestine) | 33,927 | 29,362 | 102 |
| WO18 | Adult | Fucoidan (intestine) | 42,029 | 36,201 | 76 |
| WO19 | Adult | Fucoidan (intestine) | 33,696 | 28,920 | 87 |
| WO20 | Adult | Fucoidan (intestine) | 39,691 | 33,779 | 84 |
| WO21 | Adult | Fucoidan (intestine) | 34,574 | 29,631 | 104 |
| WO22 | Adult | Fucoidan (intestine) | 36,515 | 31,132 | 104 |

Supplementary Table 3. The most abundant (>0.1 % relative abundance in total 16S rRNA gene reads obtained in this study) bacterial operational taxonomical units (OTUs) identified from larval zebrafish and from the gut of adult zebrafish

| OTU No. | Closest GenBank affiliationa | | Relative abundance (% of all 16S rRNA gene reads)[b] | | | | |
|---|---|---|---|---|---|---|---|
| | | | Larva[c] | | | Adult[d] | |
| | Taxonomy | % | control | Fuc100[e] | Fuc500[e] | control | Fuc[f] |
| OTU 1 | Escherichia/Shigella (Enterobacteriaceae) | 100 | **64.67**[g] | **66.92** | **67.45** | **35.11** | **18.00** |
| OTU 2 | Escherichia/Shigella (Enterobacteriaceae) | 100 | **17.65** | **17.63** | **17.88** | **9.33** | 4.83 |
| OTU 3 | Shinella (Rhizobiaceae) | 100 | 0.00 | 0.00 | 0.01 | **12.44** | **17.43** |
| OTU 4 | Aeromonas (Aeromonadaceae) | 100 | 0.00 | 0.01 | 0.01 | **11.87** | **14.12** |
| OTU 5 | Escherichia/Shigella (Enterobacteriaceae) | 99 | **8.37** | **8.22** | **8.16** | 4.39 | 2.24 |
| OTU 6 | Cetobacterium (Fusobacteriaceae) | 100 | 0.00 | 0.00 | 0.01 | **7.76** | **9.36** |
| OTU 7 | Shinella (Rhizobiaceae) | 100 | 0.00 | 0.00 | 0.00 | 0.55 | **8.78** |
| OTU 8 | Acidovorax (Comamonadaceae) | 100 | 0.00 | 0.00 | 0.00 | 0.04 | 7.29 |
| OTU 9 | Shewanella (Shewanellaceae) | 100 | 0.00 | 0.00 | 0.00 | 2.15 | 3.25 |
| OTU 10 | Aquabacterium (Comamonadaceae) | 100 | 0.00 | 0.05 | 0.00 | 2.51 | 2.06 |
| OTU 11 | Cetobacterium (Fusobacteriaceae) | 99 | 0.00 | 0.00 | 0.00 | 2.34 | 1.70 |
| OTU 12 | Uncultured Burkholderiales bacterium | 98 | 0.00 | 0.00 | 0.06 | 2.19 | 1.61 |
| OTU 13 | Plesiomonas (Enterobacteriaceae) | 100 | 0.00 | 0.00 | 0.00 | 1.34 | 1.04 |
| OTU 14 | Aeromonas (Aeromonadaceae) | 100 | **1.45** | 0.00 | 0.00 | 0.58 | 0.68 |
| OTU 15 | Chitinilyticum (Chromobacteriaceae) | 100 | 0.00 | 0.00 | 0.00 | 0.91 | 1.20 |
| OTU 16 | Aeromonas (Aeromonadaceae) | 100 | 0.00 | 0.00 | 0.00 | 0.76 | 0.99 |
| OTU 17 | unclassified Rhizobiales bacterium | 100 | 0.01 | 0.00 | 0.00 | 0.05 | 1.48 |
| OTU 18 | Cloacibacterium (Flavobacteriaceae) | 100 | 0.00 | 0.00 | 0.00 | 0.40 | 1.15 |
| OTU 19 | Pseudomonas (Pseudomonadaceae) | 100 | **0.65** | **0.91** | 0.48 | 0.00 | 0.00 |
| OTU 20 | Ralstonia (Burkholderiaceae) | 100 | 0.55 | **0.38** | **0.81** | 0.00 | 0.00 |
| OTU 21 | Vogesella (Chromobacteriaceae) | 99 | 0.00 | 0.00 | 0.00 | 0.55 | 0.19 |
| OTU 22 | Curvibacter (Comamonadaceae) | 99 | 0.00 | 0.12 | **0.56** | 0.00 | 0.00 |
| OTU 23 | Fluviicola (Crocinitomicaceae) | 100 | 0.00 | 0.00 | 0.00 | 0.48 | 0.01 |
| OTU 24 | Escherichia/Shigella (Enterobacteriaceae) | 100 | 0.05 | 0.07 | 0.04 | 0.18 | 0.15 |
| OTU 25 | Comamonas (Comamonadaceae) | 100 | 0.00 | 0.00 | 0.00 | 0.00 | 0.38 |
| Relative abundance of 25 OTUs in the total reads (%) | | | 93.40 | 94.31 | 95.47 | 95.93 | 97.94 |

[a] BLASTN search performed in May 2019

[b] Average % of 16S rRNA gene reads (QC passed) obtained from all samples from the same experimental conditions

[c] Bacterial 16S rRNA sequences from the whole larvae

[d] Bacterial 16S rRNA sequences from the gut of adult zebrafish

[e] Larval fish treated (immersed) with 100 µg/ml (Fuc100) or 500 µg/ml (Fuc500)

[f] Adult fish fed with OM fucoidan

[g] The five most abundant OTUs in each samples are shown in bold and highlighted
