# Supplementary material for: Intestinal Microbiota and Immune Modulation in Zebrafish by Fucoidan From Okinawa Mozuku (Cladosiphon okamuranus)
Source: Front Nutr. 2020 Jun 24;7:67. doi: 10.3389/fnut.2020.00067 (PMC7327095; doi:10.3389/fnut.2020.00067)

SUPPLEMENTARY MATERIAL

Modulation of immune responses and intestinal microbial composition in zebrafish by fucoidan derived from Okinawa *mozuku* (*Cladosiphon okamuranus*)

Wakako Ikeda-Ohtsubo[1,2*], Adrià López Nadal[2], Edoardo Zaccaria[3], Masahiko Iha[4], Haruki Kitazawa[1], Michiel Kleerebezem[3], Sylvia Brugman[2*]

Supplementary Figure Legends

Supplementary Figure S1. Alpha rarefaction curve of the total number of 16S rRNA gene sequencing from zebrafish samples.

Supplementary Figure S2. Overview of composition of bacterial groups in the larval microbiota.

Supplementary Figure S3. Overview of composition of bacterial groups in the adult intestinal microbiota.

Supplementary Figure S1

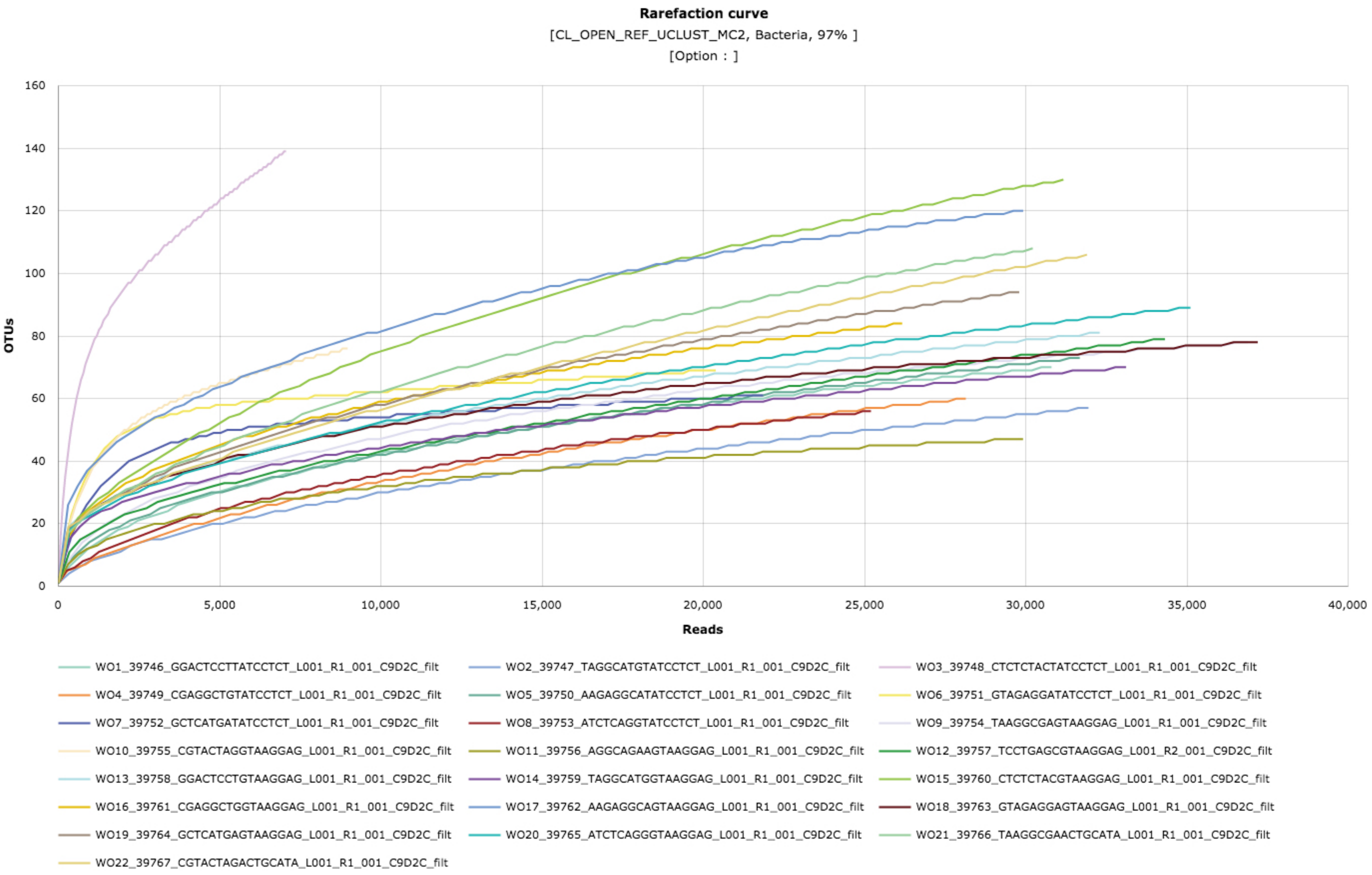

Supplementary Figure S2

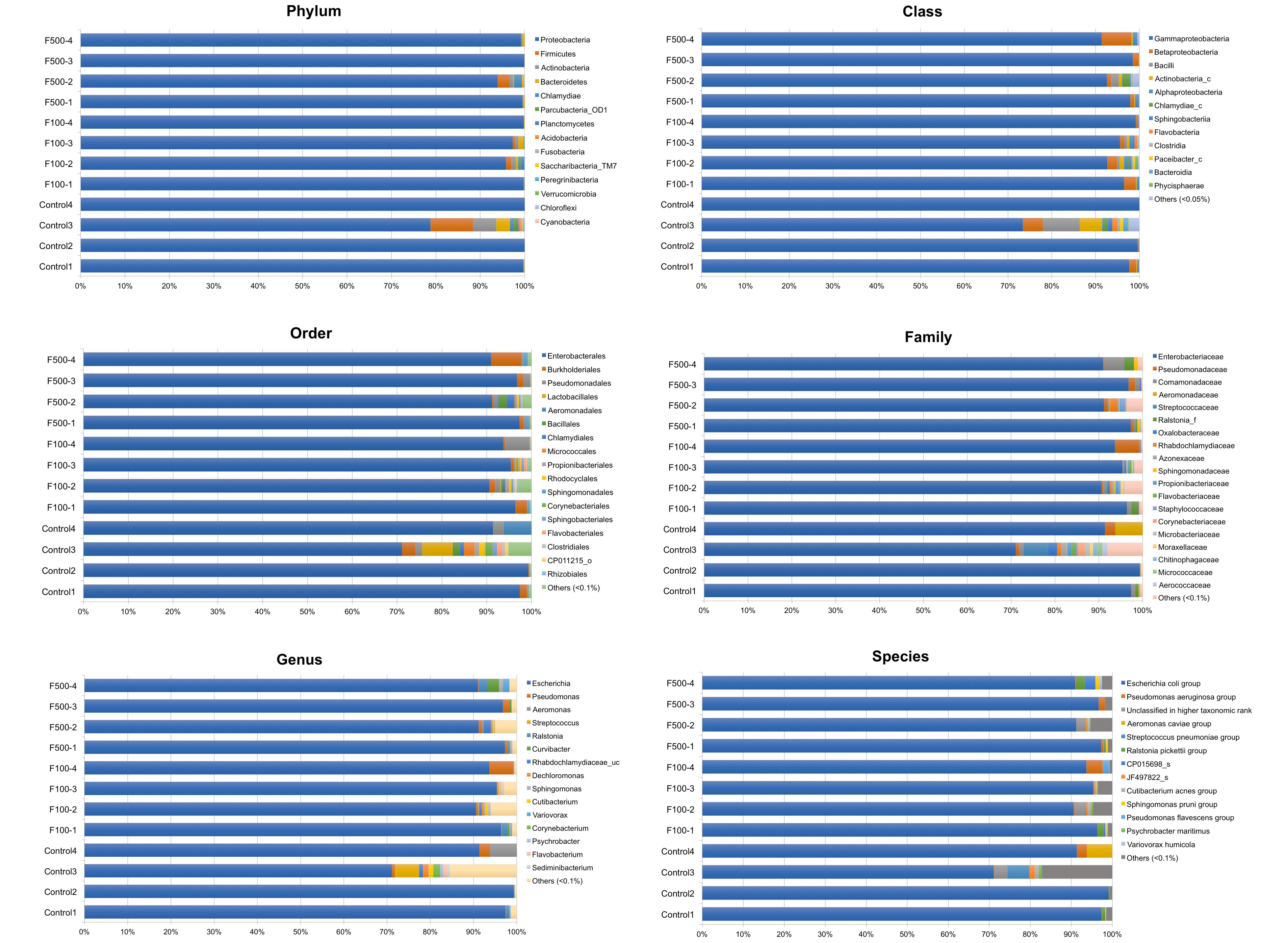

Supplementary Figure S3

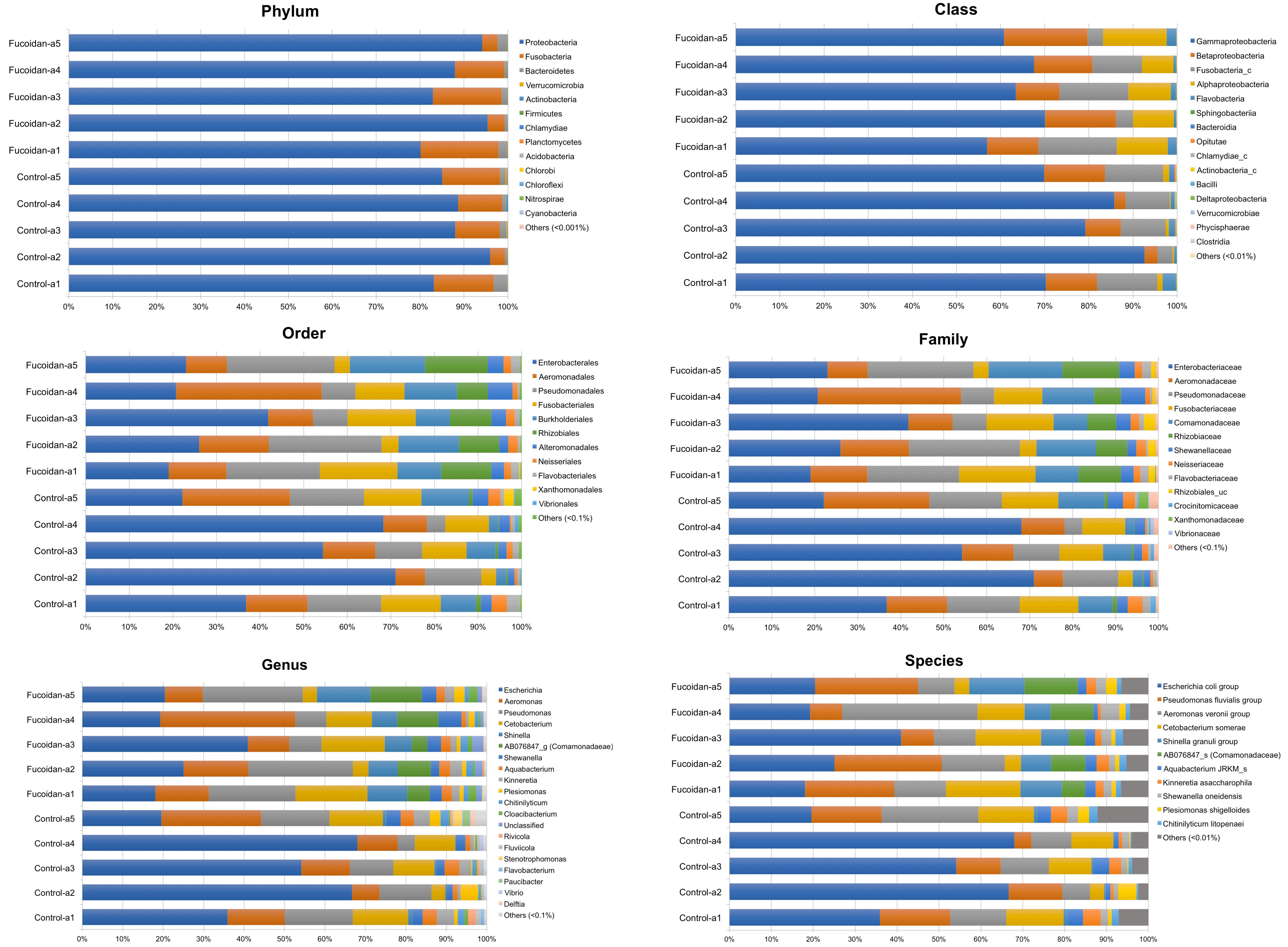

Supplement: Supplementary file 2 [file Data_Sheet_2.PDF]
